# Supplementary material for: Sequential emergence and contraction of epithelial subtypes in the prenatal human choroid plexus revealed by a stem cell model
Source: Nat Commun. 2025 Jun 3;16:5149. doi: 10.1038/s41467-025-60361-9 (PMC12134268; doi:10.1038/s41467-025-60361-9)
Supplement: Supplementary file 1 — Supplementary Information [file 41467_2025_60361_MOESM1_ESM.pdf]

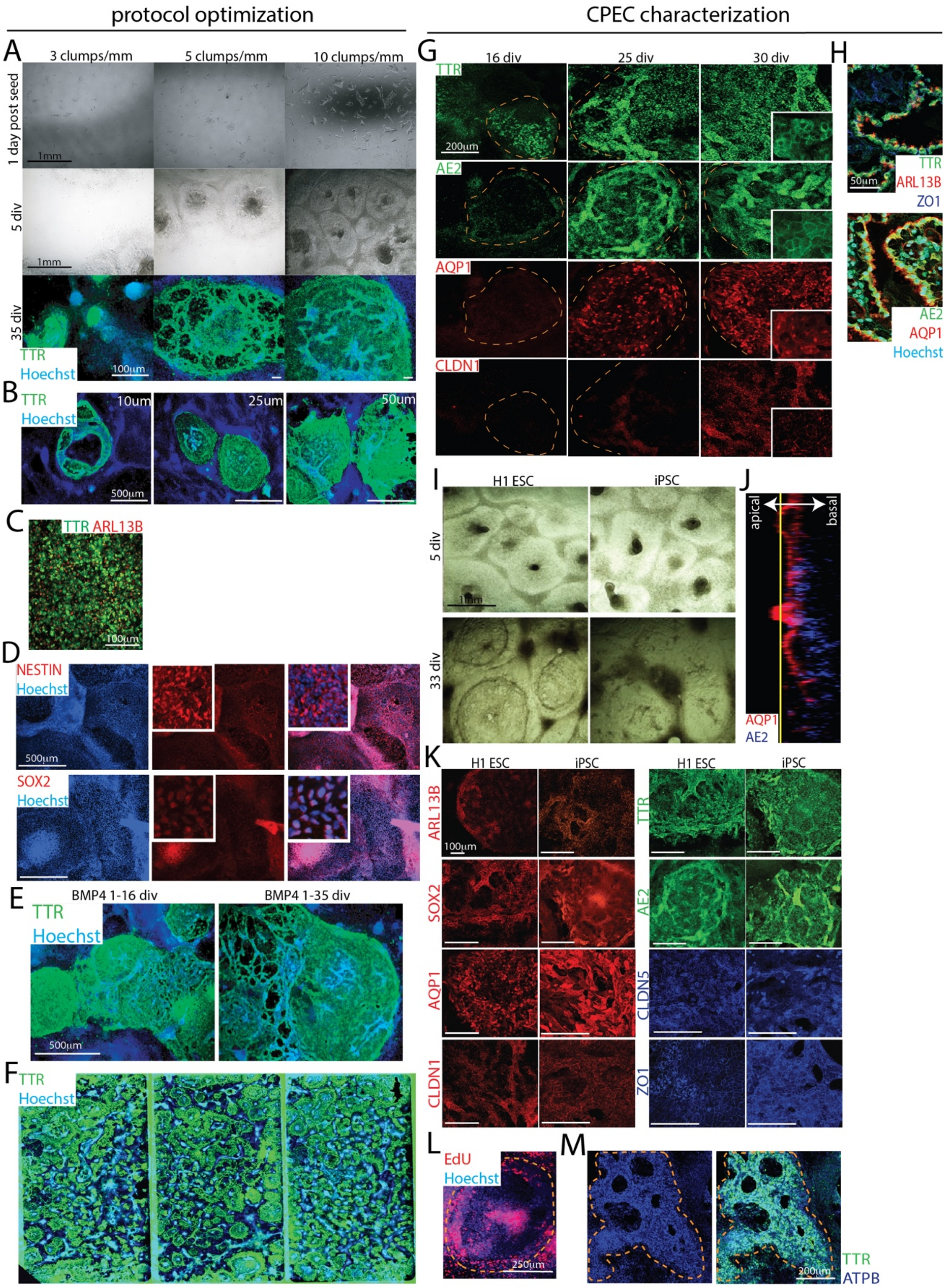
protocol optimization
CPEC characterization
A
3 clumps/mm
5 clumps/mm
10 clumps/mm
1 day post seed
5 div
35 div
1mm
TTR
Hoechst
100um
B
10um
25um
50um
500um
C
TTR ARL13B
100um
D
NESTIN
Hoechst
SOX2
500um
E
BMP4 1-16 div
BMP4 1-35 div
500um
F
G
16 div
25 div
30 div
TTR
AE2
AQP1
CLDN1
200um
H
ZO1
50um
I
H1 ESC
iPSC
33 div
J
apical
basal
K
ARL13B
SOX2
CLDN5
L
EdU
250um
M
ATPB
200um

Supplementary Figure 1: Derivation optimizations. Dashed lines demarcate dCPEC islands. Phase contrast (A,I), confocal maximal projections (H,J,M), and epifluorescent images. **(A)** Different ESC clump seeding densities (phase and TTR ICC). 10 clumps/mm^2 yields more dCPEC islands than 3 or 5 clumps/mm^2. **(B)** Different clump sizes at constant seeding density (TTR ICC, 35 div). Larger diameter clumps (50 um) yield more and larger dCPEC islands than 10 or 25 um clumps. **(C)** dCPEC induction using an alternative neural induction method (StemCell Technologies; TTR ICC, 35 div). **(D)** Uniform positivity for NESTIN and SOX2 after neural induction (ICC, 10 div). **(E)** BMP4 duration studies (TTR ICC, 35 div). No differences are apparent between 15- and 34-day applications. **(F)** Example of efficient and consistent dCPEC generation (TTR ICC, 35 div, stitched 4-well chamber slide). **(G)** Acquisition of dCPEC markers from 16-30 div (TTR, AE2, AQP1, and CLDN1 ICC). **(H)** Apicobasal polarity *in vivo* (39 pcw) with apical (ventricle-facing) ARL13B and AQP1, and basolateral AE2. **(I)** Similar phase-contrast appearance of H1 ESC and ADRC6 iPSC islands at key stages. **(J)** Uniform apicobasal polarity of ADRC6 iPSC-derived dCPECs (AQP1 and AE2 ICC, orthogonal view). **(K)** Similar CPEC marker expression in ESC- and ADRC6 iPSC-derived dCPECs (ICC, 35 div). **(L)** EdU application from 21-25 div leads to some EdU incorporation in 25-div dCPEC islands; see Fig. 1H for comparison (Click-iT reaction; Thermo Fisher). **(M)** Prominent mitochondrial staining in TTR+ dCPECs compared to non-dCPECs (ATPB ICC, 35 div).

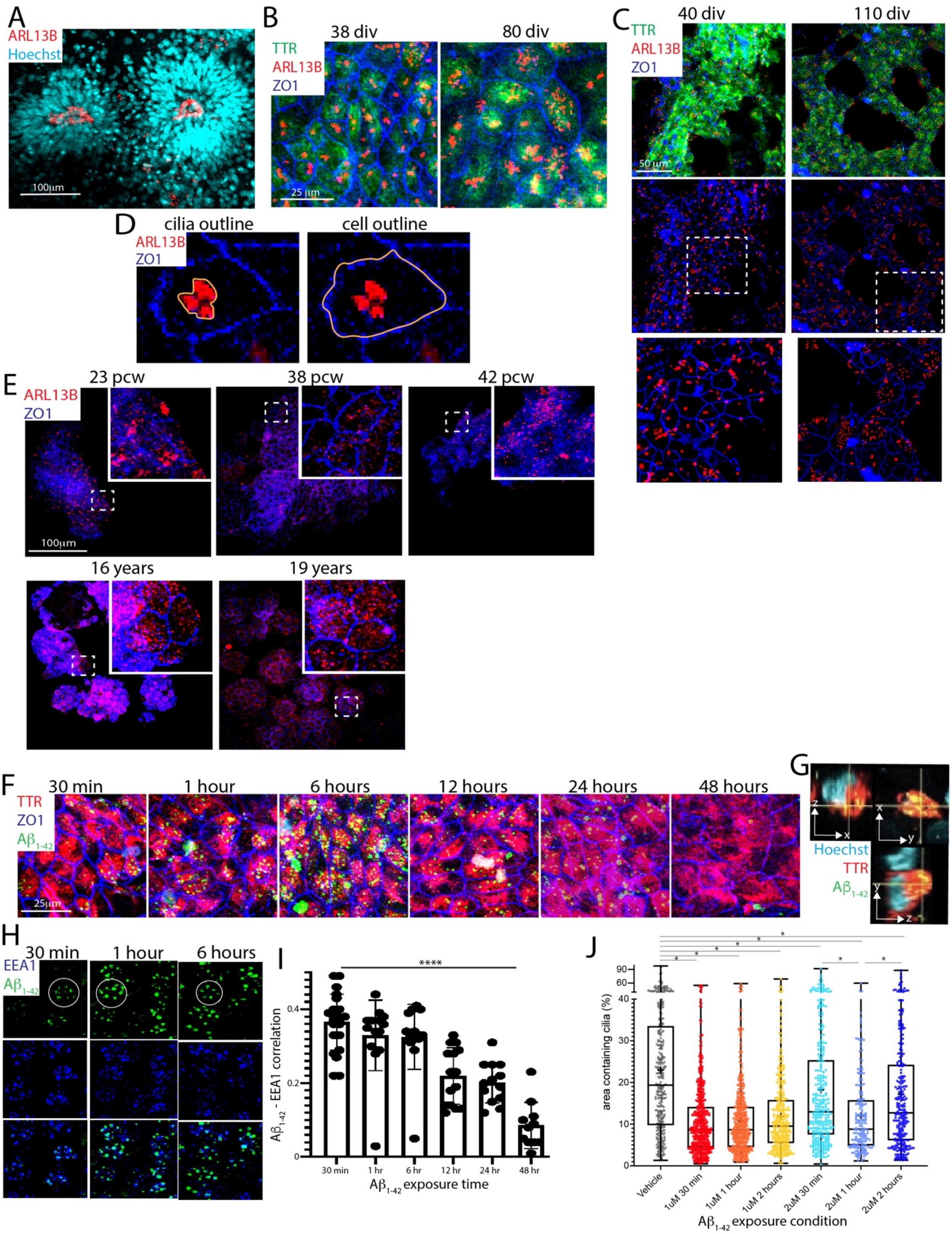

A
ARL13B
Hoechst
100μm
B
38 div
80 div
TTR
ARL13B
ZO1
25 μm
C
40 div
110 div
TTR
ARL13B
ZO1
50 μm
D
cilia outline
cell outline
ARL13B
ZO1
E
23 pcw
38 pcw
42 pcw
ARL13B
ZO1
100μm
16 years
19 years
F
30 min
1 hour
6 hours
12 hours
24 hours
48 hours
TTR
ZO1
Aβ1-42
25μm
G
Hoechst
TTR
Aβ1-42
H
30 min
1 hour
6 hours
EEA1
Aβ1-42
I
****
Aβ1-42 - EEA1 correlation
30 min
1 hr
6 hr
12 hr
24 hr
48 hr
Aβ1-42 exposure time
J
area containing cilia (%)
Vehicle
1uM 30 min
1uM 1 hour
1uM 2 hours
2uM 30 min
2uM 1 hour
2uM 2 hours
Aβ1-42 exposure condition

Supplementary Figure 2: Multiciliated and $A\beta_{1-42}$ uptake patterns in dCPECs. All images are confocal maximal projections. **(A)** ARL13B staining of neural rosettes, which is easily distinguished from dCPEC cilia staining. **(B)** Same field as Fig. 2B, but with TTR (green) to confirm dCPEC identity. **(C)** Another example of paired cultures displaying cilia clustering early (40 div) followed by dispersion (110 div). **(D)** Outlining approach for the cilia dispersion metric (ARL13B-ZO1 ICC; see Methods). **(E)** CPEC cilia staining in a more extensive *in vivo* series. ARL13B+ cilia remain dispersed in a 16- and 19-year old. **(F)** 1uM $A\beta_{1-42}$ uptake by 64-div dCPECs over a more extensive time course. **(G)** Orthogonal views displaying intracytoplasmic colocalization of $A\beta_{1-42}$ and TTR (64 div). **(H)** $A\beta_{1-42}$ in EEA1+ early endosomes (64 div). Circular $A\beta_{1-42}$ uptake patterns are apparent (circles). **(I)** Bar graph with data points (means +/- s.d.) showing decreased $A\beta_{1-42}$-EEA1 colocalization over time (one-way ANOVA $p<0.0001$****; n=12 ROIs per timepoint). **(J)** Box plots (medians, means "+", quartiles and ranges) of cilia clustering induced by 1uM or 2uM $A\beta_{1-42}$ in 64-div dCPECs (ARL13B-ZO1 ICC). All Aβ-treated cultures had dCPECs with more clustered cilia than controls by one hour (one-way ANOVA $p<0.0001$, Bonferroni corrected t-tests $p<0.05$*; vehicle n=352, 1uM 30min n=531, 1uM 1hr n=476, 1uM 2hr n=354, 2uM 30min n=294, 2uM 1hr n=184, 2uM 2hr n=267). Source data provided as a Source Data file. See statistical section of Methods for details.

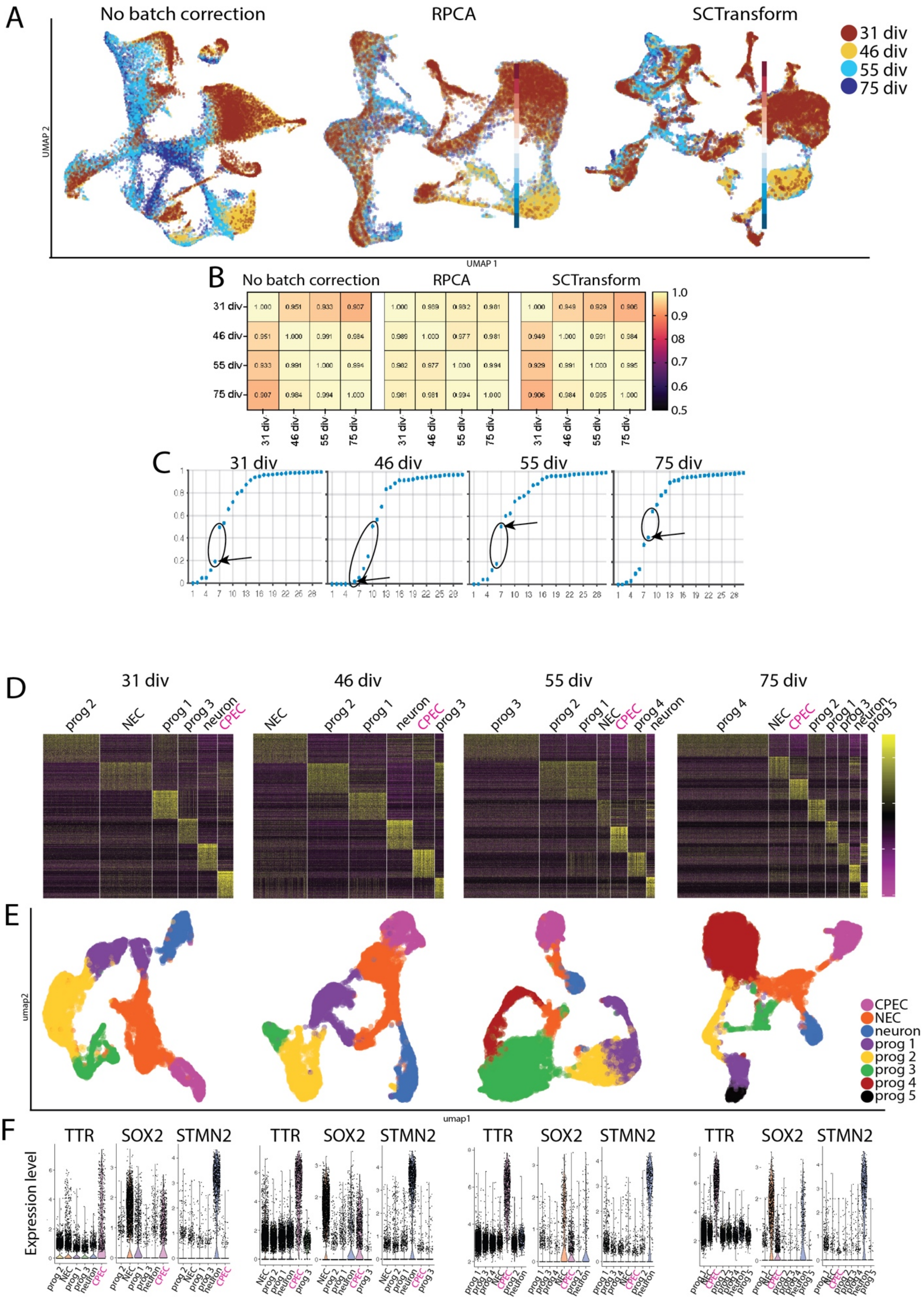
A
No batch correction
RPCA
SCTransform
31 div
46 div
55 div
75 div
UMAP 2
UMAP 1
B
No batch correction
RPCA
SCTransform
31 div
46 div
55 div
75 div
1.000 0.951 0.933 0.907 1.000 0.989 0.982 0.981 1.000 0.949 0.929 0.906
0.951 1.000 0.991 0.984 0.989 1.000 0.977 0.981 0.949 1.000 0.991 0.984
0.933 0.991 1.000 0.994 0.982 0.977 1.000 0.994 0.929 0.991 1.000 0.995
0.907 0.984 0.994 1.000 0.981 0.981 0.994 1.000 0.906 0.984 0.995 1.000
1.0
0.9
08
0.7
0.6
0.5
C
31 div
46 div
55 div
75 div
D
31 div
prog 2 NEC prog 1 prog 3 neuron CPEC
46 div
NEC prog 2 prog 1 neuron CPEC prog 3
55 div
prog 3 prog 2 prog 1 NEC CPEC prog 4 neuron
75 div
prog 4 NEC CPEC prog 2 prog 1 prog 3 neuron prog 5
E
umap2
umap1
CPEC
NEC
neuron
prog 1
prog 2
prog 3
prog 4
prog 5
F
TTR SOX2 STMN2
Expression level

Supplementary Figure 3: SoptSC quality controls and cluster number determinations (A-C) and Seurat analyses (D-F). (A) UMAPs of aggregated scRNA-seq data, color coded by timepoint, without and with RPCA or SCTransform batch correction. Clustering is discrete and spans timepoints in all cases. **(B)** Pearson correlations for dCPECs across timepoints, without and with RPCA or SCTransform batch correction. Correlations are generally high. Non-corrected and SCTransform-corrected correlations display perfect temporal ordering. Color scale reflects r values 0-1. **(C)** SoptSC cluster number determinations by Eigengap heuristic; ranges of bioinformatically-supported clusters are circled. Lowest cluster numbers were selected (arrows), except for 55 div, which yielded excellent DEG heatmaps using the higher number, as in D. **(D)** Heatmaps of top-100 DEGs support Eigengap heuristic-based cluster numbers. Color scale of Z-score of DEGs. **(E)** Seurat UMAPs supporting SoptSC-determined cluster numbers and the direct origin of dCPECs (purple) from NECs (orange). **(F)** Violin plots of cell-type marker gene expression using Seurat-generated clusters. TTR highlights dCPECs, SOX2 highlights NECs, and STMN2 highlights neurons.

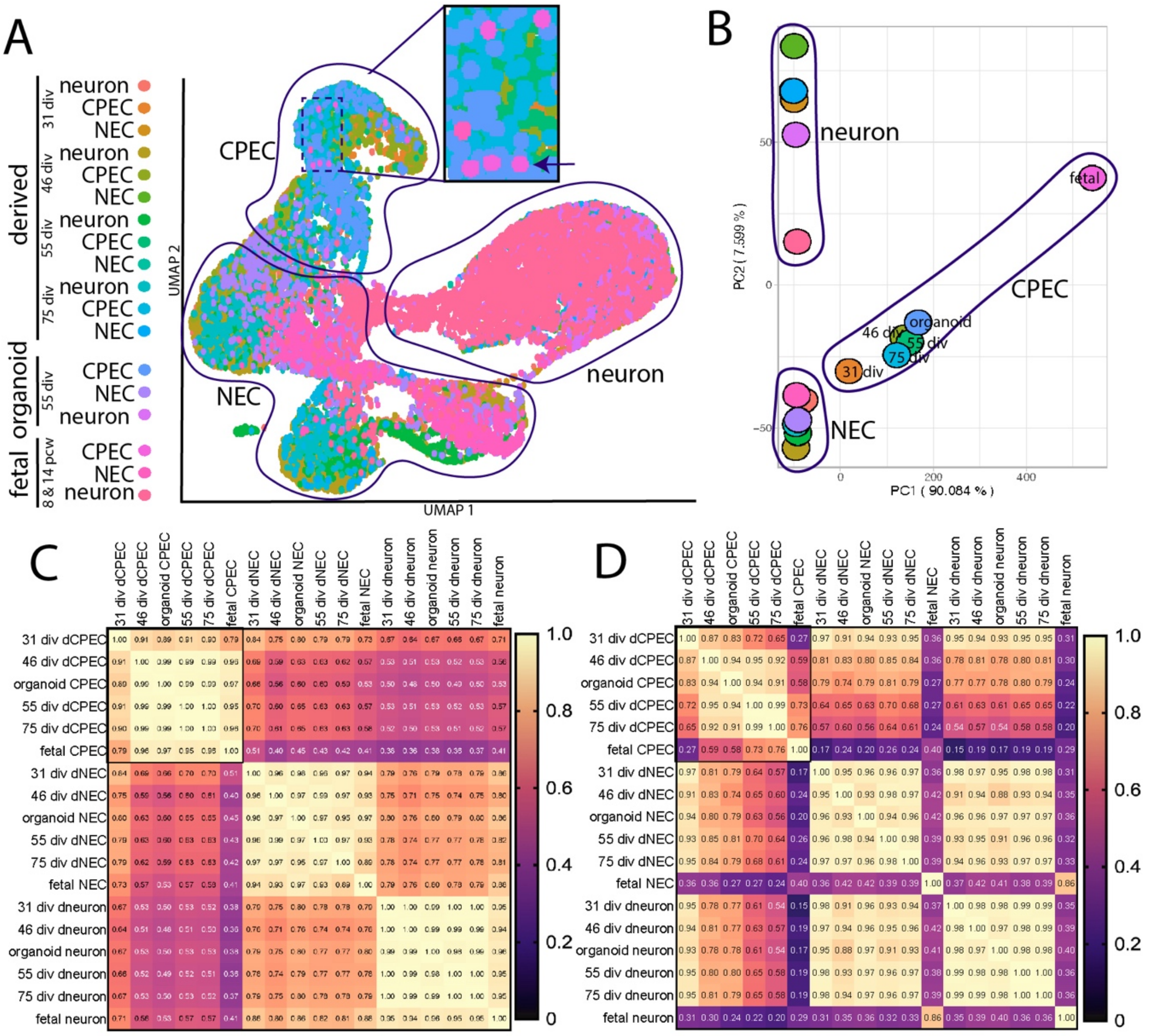
A
derived
31 div
46 div
55 div
75 div
organoid
55 div
fetal
8 & 14 pcw
neuron
CPEC
NEC
UMAP 1
UMAP 2
CPEC
NEC
neuron
B
PC1 (90.084 %)
PC2 (7.599 %)
neuron
CPEC
NEC
fetal
organoid
46 div
55 div
75 div
31 div
C
D
31 div dCPEC
46 div dCPEC
organoid CPEC
55 div dCPEC
75 div dCPEC
fetal CPEC
31 div dNEC
46 div dNEC
organoid NEC
55 div dNEC
75 div dNEC
fetal NEC
31 div dneuron
46 div dneuron
organoid neuron
55 div dneuron
75 div dneuron
fetal neuron
1.0
0.8
0.6
0.4
0.2
0

Supplementary Figure 4: Additional dCPEC, organoid CPEC, and fetal CPEC comparisons. (A) RPCA-corrected UMAP of aggregated datasets. CPECs, NECs, and neurons from each dataset cluster well (outlined). Within the CPEC cluster, fetal CPECs (pink, arrow in inset) are more similar to older dCPECs (green-blue). **(B)** RPCA-corrected PCA of aggregated datasets, with CPECs, NECs, and neurons outlined. **(C)** RPCA-corrected Pearson correlation table for CPECs (boxed upper left), NECs, and neurons (lower right). Correlations between CPECs from the three datasets are high, as they are for NECs and neurons. **(D)** SCTransform-corrected Pearson correlation table, organized as in C. Correlations among the CPECs are generally lower, while NECs and neurons appear more similar. Color scale reflects r values 0-1.

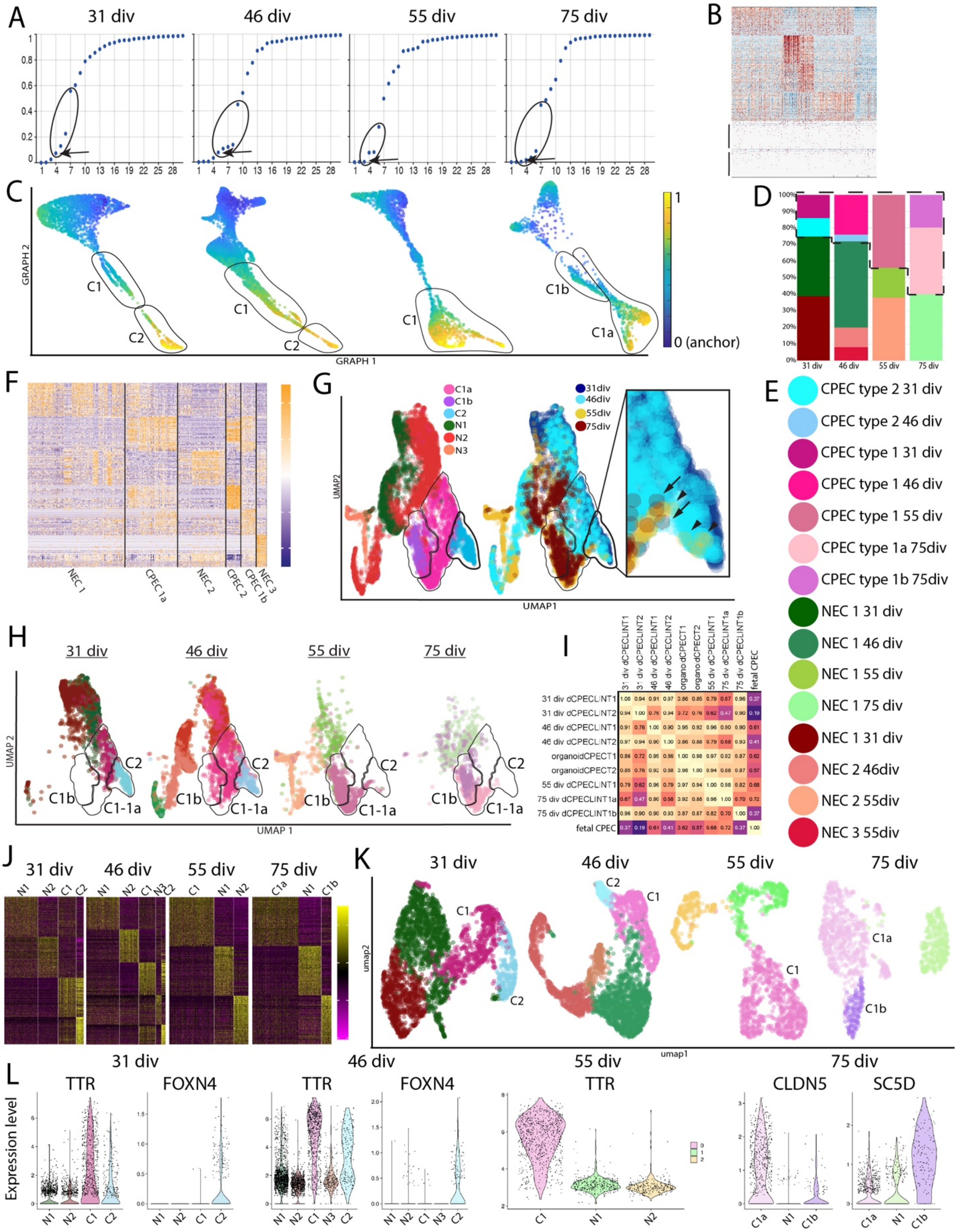
A
31 div
46 div
55 div
75 div
B
C
C1
C2
C1b
C1a
GRAPH 2
GRAPH 1
1
0 (anchor)
D
31 div
46 div
55 div
75 div
E
CPEC type 2 31 div
CPEC type 2 46 div
CPEC type 1 31 div
CPEC type 1 46 div
CPEC type 1 55 div
CPEC type 1a 75div
CPEC type 1b 75div
NEC 1 31 div
NEC 1 46 div
NEC 1 55 div
NEC 1 75 div
NEC 1 31 div
NEC 2 46div
NEC 2 55div
NEC 3 55div
F
NEC 1
CPEC 1a
NEC 2
CPEC 2
CPEC 1b
NEC 3
G
C1a
C1b
C2
N1
N2
N3
31div
46div
55div
75div
UMAP2
UMAP1
H
31 div
46 div
55 div
75 div
C2
C1b
C1-1a
UMAP 2
UMAP 1
I
31 div dCPECLINT1
31 div dCPECLINT2
46 div dCPECLINT1
46 div dCPECLINT2
organoidCPECT1
organoidCPECT2
55 div dCPECLINT1
75 div dCPECLINT1a
75 div dCPECLINT1b
fetal CPEC
J
31 div
46 div
55 div
75 div
K
31 div
46 div
55 div
75 div
C1
C2
C1a
C1b
umap2
umap1
L
31 div
46 div
55 div
75 div
TTR
FOXN4
CLDN5
SC5D
Expression level
N1
N2
N3
C1
C2
C1a
C1b

Supplementary Figure 5: Additional SoptSC (A-I) and Seurat analyses (J-L) of dCPEC subtypes. (A) Eigengap heuristic for aggregated dCPECs and NECs. Ranges of bioinformatically-supported clusters are circled. The lowest cluster numbers were selected (arrows). **(B)** DEG heatmap example of overclustering. A 31-div heatmap for 6 clusters includes two without distinctive signatures (lower rows) and supporting 4 as the more appropriate choice. **(C)** Pseudotemporal color coding of SoptSC GRAPH charts shown in Fig. 4B, with NECs as anchor. NECs are more similar to C1 than C2 cells at 31 and 46 div. Both C1a and C1b cells have similarities to NECs. **(D-E)** Stacked bar graphs of dCPEC and NEC subtypes, with color coding in E. Total dCPEC and C1 fractions increase over time compared to NECs. **(F)** Heatmap of top-100 dCPEC and NEC DEGs aggregated across timepoints, which suggests 3 dCPEC and 3 NEC clusters like the individual timepoint analyses in Fig. 4A. Color scale of Z-score of DEGs. **(G)** UMAPs of aggregated dCPECs and NECs, color coded by subtype (left) or timepoint (right), with dCPEC subtype regions outlined. Some C2 cells at 55 div (arrowheads) and 75 div (arrows) are detected. **(H)** UMAPs per timepoint, as in Fig. 4E, plus NEC subtypes. Similar dCPEC patterns are seen, including C1 lineage adoption of a hybrid C1a-C1b profile at 55 div before bifurcating by 75 div. **(I)** SCTransform-corrected Pearson correlations for organoid CPECs, fetal CPECs, and dCPECs separated by subtype and timepoint. 75-div C1a dCPECs are more similar than C1b cells to 55-div C1 dCPECs. **(J)** Seurat heatmaps of top-100 DEGs, supporting dCPEC and NEC cluster numbers and designations based on SoptSC. Color scale of Z-score of DEGs. **(K)** Seurat UMAPs supporting dCPEC and NEC cluster numbers and designations based on SoptSC. **(L)** Violin plots of dCPEC subtype marker expression in Seurat-generated clusters.

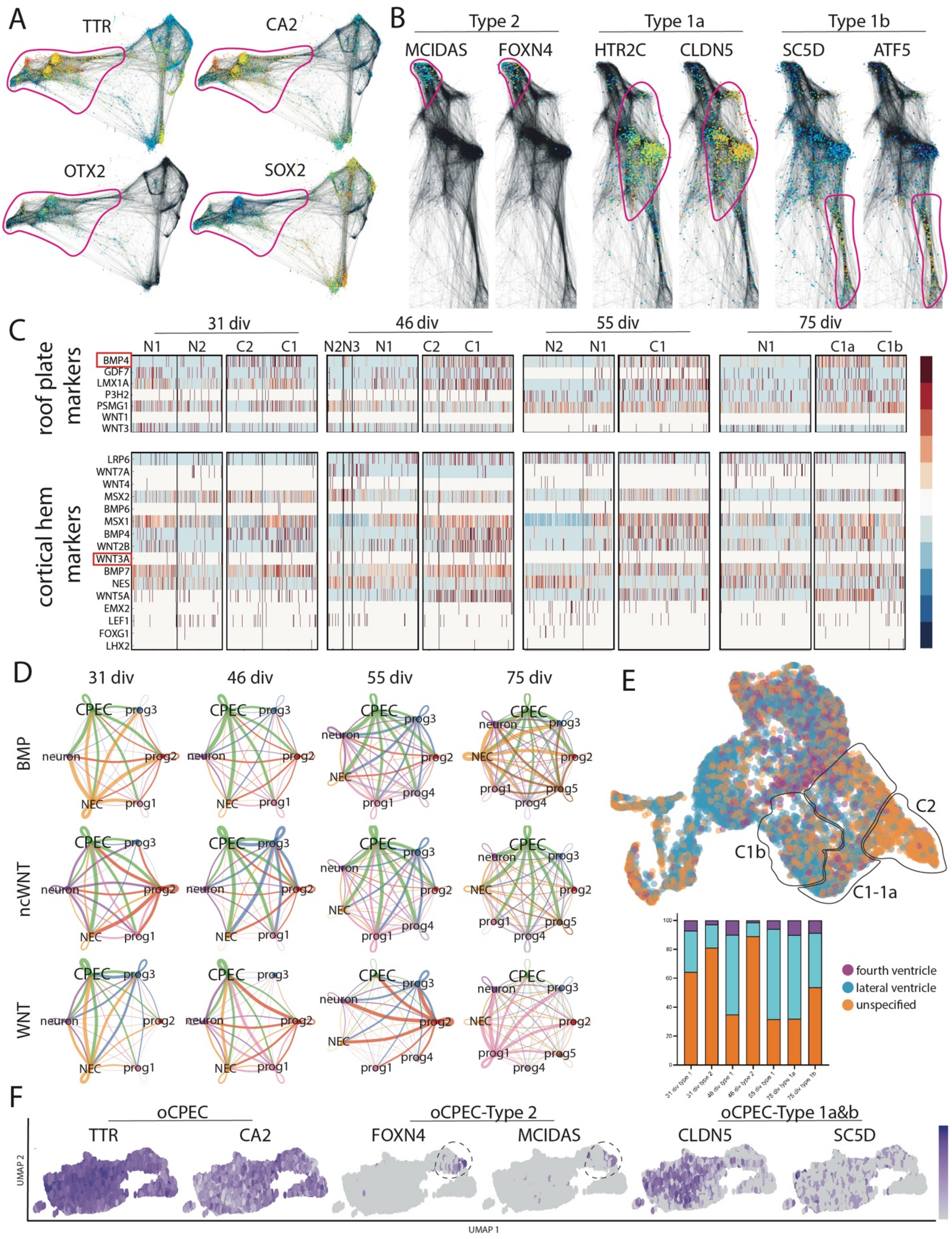
A
TTR
CA2
OTX2
SOX2
B
Type 2
Type 1a
Type 1b
MCIDAS
FOXN4
HTR2C
CLDN5
SC5D
ATF5
C
31 div
46 div
55 div
75 div
N1 N2 C2 C1
N2N3 N1 C2 C1
N2 N1 C1
N1 C1a C1b
roof plate markers
BMP4
GDF7
LMX1A
P3H2
PSMG1
WNT1
WNT3
cortical hem markers
LRP6
WNT7A
WNT4
MSX2
BMP6
MSX1
BMP4
WNT2B
WNT3A
BMP7
NES
WNT5A
EMX2
LEF1
FOXG1
LHX2
D
31 div
46 div
55 div
75 div
BMP
ncWNT
WNT
CPEC
prog1
prog2
prog3
prog4
prog5
neuron
NEC
E
C2
C1b
C1-1a
fourth ventricle
lateral ventricle
unspecified
F
oCPEC
oCPEC-Type 2
oCPEC-Type 1a&b
TTR
CA2
FOXN4
MCIDAS
CLDN5
SC5D
UMAP 2
UMAP 1

Supplementary Figure 6: Additional bioinformatic analyses, including STITCH and CellChat. **(A)** STITCH dot plots of aggregated dCPECs and NECs, as in Fig. 4F-H, highlighting CPEC (TTR, CA2, OTX2) and NEC markers (SOX2); dCPEC regions outlined. **(B)** STITCH dot plots of aggregated dCPECs, highlighting type 2 (MCIDAS, FOXN4), type 1a (HTR2C, CLDN5), and type 1b markers (SC5D, ATF5); subtype regions outlined. **(C)** Heatmap of roof plate (top) and cortical hem (bottom) markers per timepoint. Patterns suggestive of unique relationships to dCPECs or dCPEC subtypes are not seen. Among BMPs, BMP4 and BMP7 are expressed by dCPECs, while WNT2B and WNT5A are expressed among WNTs. Color scale of Z-score of DEGs. **(D)** CellChat analyses of major cell types for BMP, non-canonical WNT (ncWNT), and WNT signaling per timepoint. Predicted autocrine dCPEC signaling (green loops) and dCPEC signaling to other cell types (green curves) are prominent. **(E)** Ventricular (regional) specification of aggregated dCPECs and NECs using mouse genes[11]. Lateral (blue) exceeds 4th ventricular identities (purple), but identities are mixed, include substantial unspecified fractions, and do not distinguish dCPEC subtypes. **(F)** Organoid CPEC[13] (oCPEC) expression of dCPEC subtype markers. Organoid CPECs express pan-CPEC markers (left) and include a C2 subpopulation (circled in middle), but C1a ($CLDN5^{hi}SC5D^{lo}$) and C1b ($SC5D^{hi}CLDN5^{lo}$) subpopulations are not distinguished. Color scale reflects the log normalized expression levels of the markers where white is zero and the dark purple reflects the highest expression level for each of the markers. Source data provided as a Source Data file.

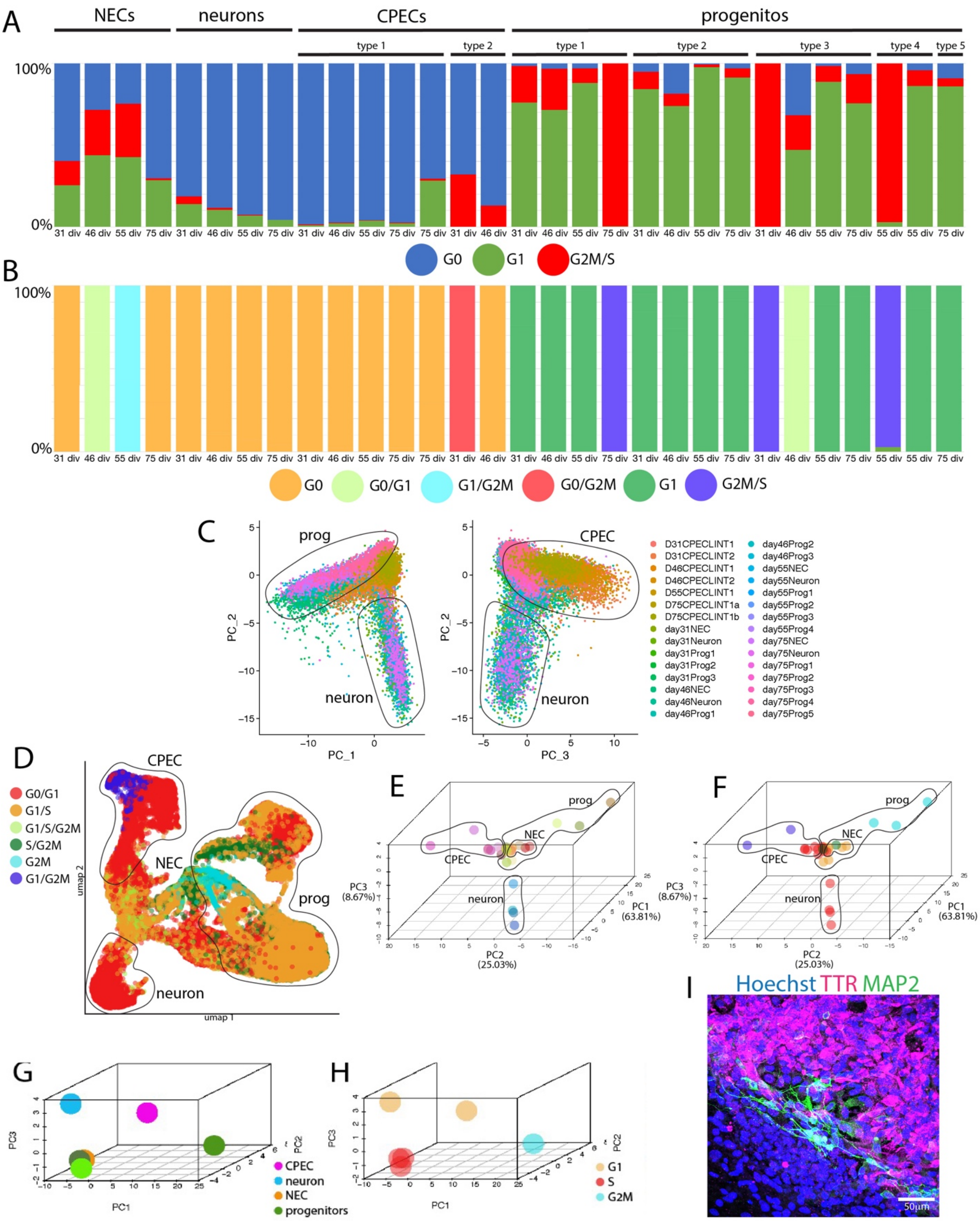
A
NECs
neurons
CPECs
progenitos
type 1
type 2
type 1
type 2
type 3
type 4
type 5
100%
0%
31 div
46 div
55 div
75 div
G0
G1
G2M/S
B
G0
G0/G1
G1/G2M
G0/G2M
G1
G2M/S
C
prog
neuron
CPEC
PC_1
PC_2
PC_3
D31CPECLINT1
D31CPECLINT2
D46CPECLINT1
D46CPECLINT2
D55CPECLINT1
D75CPECLINT1a
D75CPECLINT1b
day31NEC
day31Neuron
day31Prog1
day31Prog2
day31Prog3
day46NEC
day46Neuron
day46Prog1
day46Prog2
day46Prog3
day55NEC
day55Neuron
day55Prog1
day55Prog2
day55Prog3
day55Prog4
day75NEC
day75Neuron
day75Prog1
day75Prog2
day75Prog3
day75Prog4
day75Prog5
D
CPEC
NEC
prog
neuron
G0/G1
G1/S
G1/S/G2M
S/G2M
G2M
G1/G2M
umap 1
umap 2
E
F
prog
NEC
CPEC
neuron
PC3 (8.67%)
PC1 (63.81%)
PC2 (25.03%)
I
Hoechst TTR MAP2
50µm
G
H
PC1
PC2
PC3
CPEC
neuron
NEC
progenitors
G1
S
G2M

Supplementary Figure 7: Additional cell cycle analyses using 'neural G0' (A-C) and Seurat (D-H). (A) Stacked bar graphs color coded by neural G0 phase (G0, G1, G2M/S). In contrast to NECs and progenitors, dCPEC subtypes have predominant G0 signatures (blue) and lower G1 fractions (green) than the neurons. 75-div C1b cells are also G0-predominant, but have a higher G1 fraction than other dCPEC subtypes. Some C2 cells have a G2M/S signature (red) due to shared ciliogenesis and M-phase genes. **(B)** Color coding for subtype clusters used in Fig. 5D. Clusters are assigned to their predominant phase unless a second phase exceeds 30%, in which case a cluster is designated and color coded for "dual" phases. **(C)** 2D PCA scatterplots using neural G0 genes. The second PC distinguishes the neurons (left) while the third PC distinguishes the dCPECs (right). **(D)** UMAP as in Fig. 5, but using Seurat cell cycle genes (mouse), color coded for cell cycle phase. The dCPECs and neurons have predominant G0/G1 signatures (red). G1/G2M C2 cells are also evident (blue). **(E-F)** 3D PCA plot of subtype clusters using Seurat cell cycle genes, color coded for subtype or cell cycle phase as in C or D, respectively. The three branches of NEC progeny are evident. **(G-H)** 3D PCA plot of 31-div cell-type clusters using Seurat cell cycle genes, color coded for cell type (G) or cell cycle phase (H). G0/G1 dCPEC and neuron clusters are distinct. **(I)** The same field as in Fig. 5G, without KI67 and with Hoechst, to enable non-dCPEC estimates outside the dCPEC island.

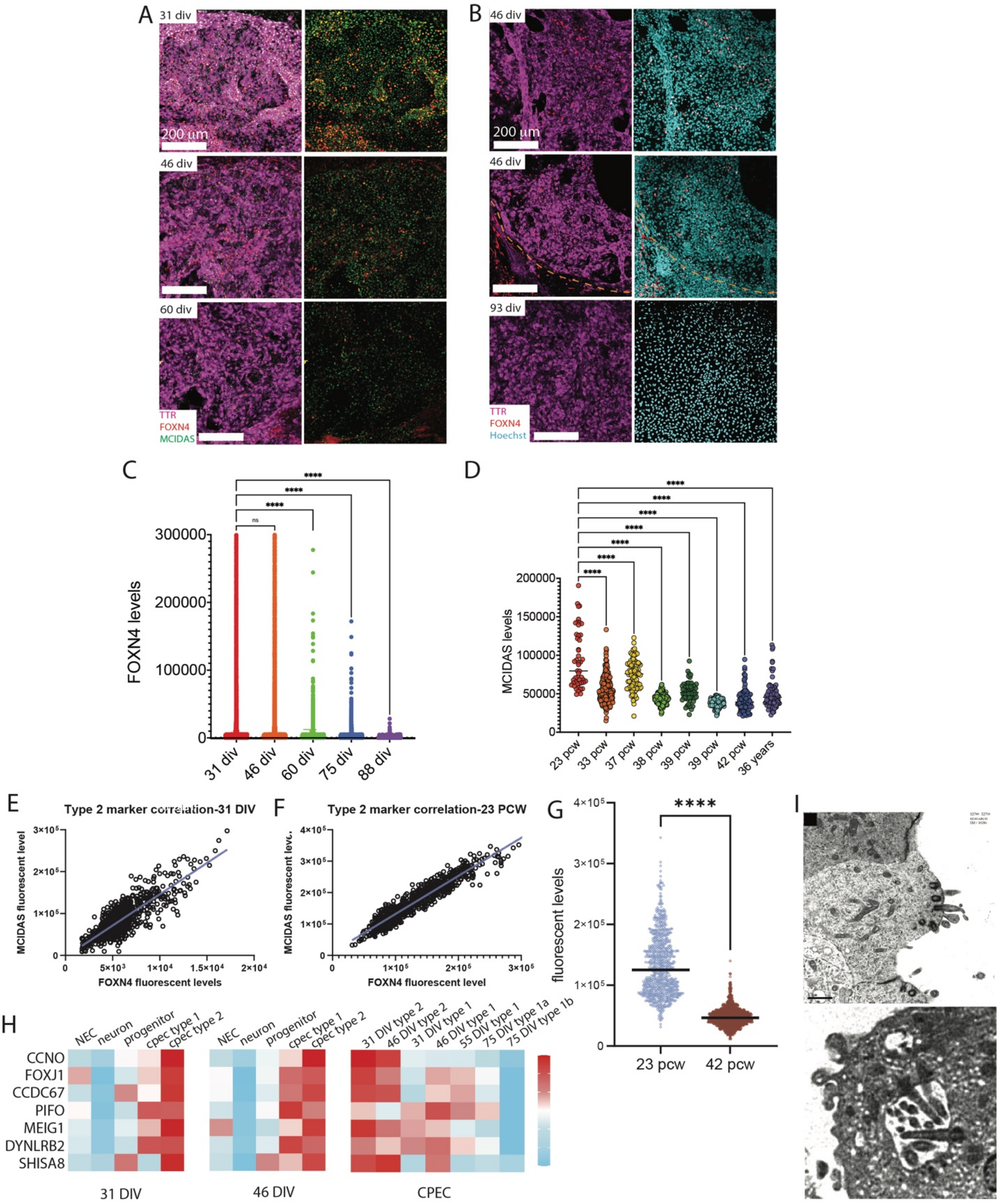

A
31 div
200 µm
46 div
60 div
TTR
FOXN4
MCIDAS
B
46 div
200 µm
46 div
93 div
TTR
FOXN4
Hoechst
C
FOXN4 levels
300000
200000
100000
0
31 div
46 div
60 div
75 div
88 div
ns
****
D
MCIDAS levels
200000
150000
100000
50000
0
23 pcw
33 pcw
37 pcw
38 pcw
39 pcw
39 pcw
42 pcw
36 years
****
E
Type 2 marker correlation-31 DIV
MCIDAS fluorescent level
FOXN4 fluorescent levels
F
Type 2 marker correlation-23 PCW
FOXN4 fluorescent level
G
fluorescent levels
23 pcw
42 pcw
****
H
NEC
neuron
progenitor
cpec type 1
cpec type 2
CCNO
FOXJ1
CCDC67
PIFO
MEIG1
DYNLRB2
SHISA8
31 DIV
46 DIV
31 DIV type 2
46 DIV type 2
31 DIV type 1
46 DIV type 1
55 DIV type 1
75 DIV type 1a
75 DIV type 1b
CPEC
I

Supplementary Figure 8: Additional studies on early type 1 and type 2 dCPECs. All images are confocal maximal projections. **(A)** Type 2 dCPECs in parallel cultures (FOXN4-MCIDAS-TTR ICC; 31, 46, and 60 div). FOXN4 (red) and MCIDAS (green) fractional positivities and expression levels decrease over time. **(B)** Lower-power views of the FOXN4 studies shown in Fig. 6F (FOXN4-TTR ICC; 46 and 93 div), dashed lines demarcate dCPEC islands. In addition to the decreasing FOXN4+ C2 fraction, a TTR-negative non-dCPEC population stains for FOXN4 (middle panel). **(C)** Expanded *in vitro* series of parallel cultures (31-88 div) demonstrating type 2 contraction over time (FOXN4 ICC). **(D)** Expanded *in vivo* series of choroid plexus tissues (23 pcw-36 years) demonstrating type 2 contraction after midgestation (23 pcw) (MCIDAS IHC). **(E)** Positive linear Pearson correlation of FOXN4 and MCIDAS fluorescence levels in 31-div dCPECs ($r=0.8308$, $p<0.0001$; $n=1003$). **(F)** Positive linear Pearson correlation of FOXN4 and MCIDAS fluorescence levels in 23 pcw ChP tissue ($r=0.9486$, $p<0.0001$; $n=799$). **(G)** Violin plots with medians of FOXN4 fluorescence levels in CPECs *in vivo*, which decrease between 23 and 42 pcw (t-test $p<0.0001$****; 23-pcw $n=799$, 42-pcw $n=1504$). **(H)** Heatmap of C2 markers, including master ciliogenesis genes CCNO and FOXJ1. Compensation for the reductions in ciliogenic cells and gene enrichment is not evident at later *in vitro* stages. Color scale of Z-score of DEGs. **(I)** Transmission electron micrographs at 40 div demonstrating dCPECs with multiple basal bodies (upper image) and a ciliary pocket with multiple cilia (lower image). Note also the 'dark' dCPEC immediately adjacent to the 'light' dCPEC with multiple basal bodies (upper image). Source data provided as a Source Data file. See statistical section of Methods for details.

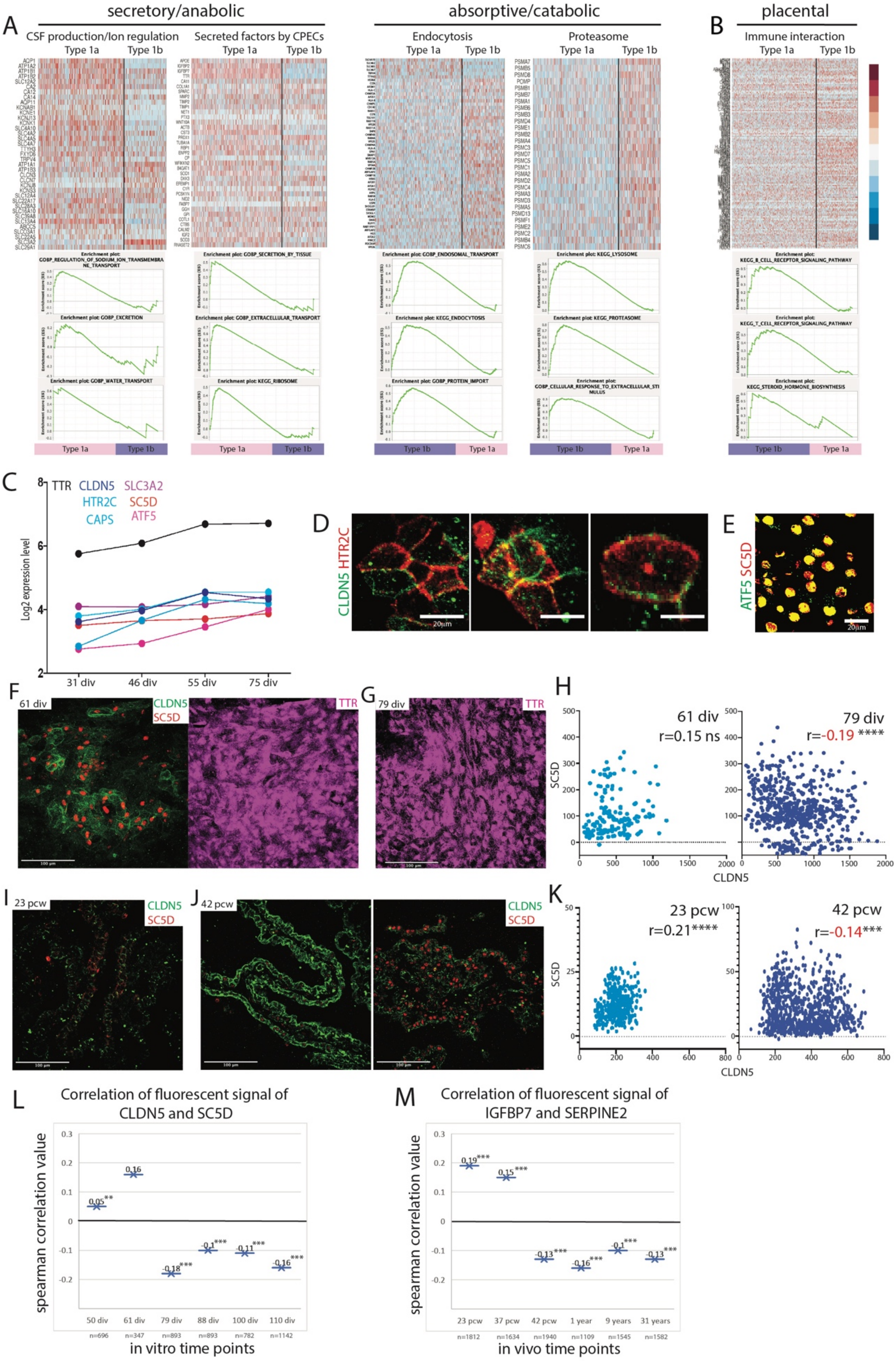

A
secretory/anabolic
absorptive/catabolic
B
placental
CSF production/Ion regulation
Secreted factors by CPECs
Endocytosis
Proteasome
Immune interaction
Type 1a
Type 1b
C
TTR
CLDN5
SLC3A2
HTR2C
SC5D
CAPS
ATF5
Log2 expression level
31 div
46 div
55 div
75 div
D
CLDN5 HTR2C
E
ATF5 SC5D
F
61 div
G
79 div
TTR
H
r=0.15 ns
r=-0.19 ****
SC5D
CLDN5
I
23 pcw
J
42 pcw
K
r=0.21****
r=-0.14***
L
Correlation of fluorescent signal of CLDN5 and SC5D
spearman correlation value
in vitro time points
50 div
61 div
79 div
88 div
100 div
110 div
M
Correlation of fluorescent signal of IGFBP7 and SERPINE2
in vivo time points
23 pcw
37 pcw
1 year
9 years
31 years

Supplementary Figure 9: Additional studies on late type 1a and type 1b dCPECs. (A) Secretory/anabolic (left) and absorptive/catabolic pathways (right) enriched in C1a and C1b cells, respectively. (Upper) Heatmaps of manually-curated secretory/anabolic (CSF production/ion regulation, secreted factors) and absorptive/catabolic genes (endocytosis, proteasome). (Lower) GSEA enrichment plots for secretory/anabolic (regulation of sodium ion transmembrane transport, excretion, water transport, secretion, extracellular transport, ribosome) and absorptive/catabolic pathways (endosomal transport, endocytosis, protein import, lysosome, proteasome, cellular response to extracellular stimulus). **(B)** Placental functions[86] enriched in C1b cells. Heatmaps and GSEA enrichment plots for immune interaction genes, B-cell receptor signaling, T-cell receptor signaling, and steroid hormone biosynthesis. Color scale of DEGs Z-score. **(C)** Expression levels in individual genes used for pairwise correlations in Fig. 7D (C1a markers: CLDN5, HTR2C, CAPS; C1b markers: SLC3A2, SC5D, ATF5). **(D)** ICC validation of CLDN5 as a C1a marker based on membrane colocalization with HTR2C, another C1a marker (75-div). **(E)** ICC validation of SC5D as a C1b marker based on nuclear colocalization with ATF5, another C1b marker (75-div). **(F-H)** C1a-C1b specification *in vitro* (CLDN5-SC5D ICC maximal projections and scatterplots with Pearson correlations; 61- and 79-div). TTR+ dCPECs at 61-div displaying positively-correlated CLDN5-SC5D expression (F); TTR positivity of the 79-div field shown in Fig. 7F-G (G); scatterplots of CLDN5-SC5D coexpression in individual dCPECs, whose correlation trends positively at 61-div, then becomes significantly negative by 79-div (H) ($p=0.15^{ns}$, n=150; p<0.0001****, n=584). **(I-K)** C1a-C1b specification *in vivo* (CLDN5-SC5D IHC maximal projections and scatterplots with Pearson correlations; 23- and 42-pcw). Relatively low and positively-correlated CLDN5-SC5D expression at 23-pcw (I); relatively large fields of $CLDN5^{hi}$-$SC5D^{lo}$ (C1a) and $SC5D^{hi}$-$CLDN5^{lo}$ (C1b) cells at 42-pcw (J); scatterplots of CLDN5-SC5D coexpression in CPECs *in vivo*, whose correlation is significantly positive at 23-pcw, then becomes significantly negative by 42-pcw (p<0.0001****, n=323; p<0.001***, n=812). **(L)** Expanded *in vitro* series (50-110-div) of CLDN5-SC5D Spearman correlations highlighting C1a-C1b differentiation between 61- and 79-div. **(M)** Expanded *in vivo* series

(23-pcw-31-years) of Spearman correlations using independent cytoplasmic markers for C1a (IGFBP7) and C1b (SERPINE2) highlighting C1a-C1b differentiation near term. Source data provided as a Source Data file. See statistical section of Methods for details.
