# Supplementary material for: Sequential emergence and contraction of epithelial subtypes in the prenatal human choroid plexus revealed by a stem cell model
Source: Nat Commun. 2025 Jun 3;16:5149. doi: 10.1038/s41467-025-60361-9 (PMC12134268; doi:10.1038/s41467-025-60361-9)
Supplement: Supplementary file 2 — Description of Additional Supplementary Information [file 41467_2025_60361_MOESM2_ESM.docx]

**Description of Additional Supplementary Files**

File Name: Supplementary Data 1

Description: Lists of DEGs and enriched pathways in scRNA-seq clusters. Each tab shows the genes/pathways associated with a different cluster. Information on postmortem patient samples is provided in the last tab.

File Name: Supplementary Movie 1

Description: 1x time-lapse DIC imaging using a 60x objective of a 42-div dCPEC island, focused on the crater-like edge of the island. Cilia in profile project from dCPEC apical surfaces and can move rapidly.

File Name: Supplementary Movie 2

Description: Similar to Supplementary Movie 1, but focused on the crater-like floor of the dCPEC island. Cilia in profile and en face are more readily visible and can move rapidly. Variable patterns of movement are seen.

File Name: Supplementary Movie 3

Description: 1x time-lapse DIC imaging using a 60x objective of 110-div dCPECs. Cilia within the focal plane are visible (circled in red), but display little to no movement.
