# Supplementary material for: Sequential emergence and contraction of epithelial subtypes in the prenatal human choroid plexus revealed by a stem cell model
Source: Nat Commun. 2025 Jun 3;16:5149. doi: 10.1038/s41467-025-60361-9 (PMC12134268; doi:10.1038/s41467-025-60361-9)
Supplement: Supplementary file 7 — Reporting Summary [file 41467_2025_60361_MOESM7_ESM.pdf]

nature portfolio

Corresponding author(s): Edwin S. Monuki

Last updated by author(s): 05/15/2025

Reporting Summary

Nature Portfolio wishes to improve the reproducibility of the work that we publish. This form provides structure for consistency and transparency in reporting. For further information on Nature Portfolio policies, see our Editorial Policies and the Editorial Policy Checklist.

Please do not complete any field with "not applicable" or n/a. Refer to the help text for what text to use if an item is not relevant to your study.
For final submission: please carefully check your responses for accuracy; you will not be able to make changes later.

Statistics

For all statistical analyses, confirm that the following items are present in the figure legend, table legend, main text, or Methods section.

n/a	Confirmed	
☐	☒	The exact sample size (*n*) for each experimental group/condition, given as a discrete number and unit of measurement
☐	☒	A statement on whether measurements were taken from distinct samples or whether the same sample was measured repeatedly
☐	☒	The statistical test(s) used AND whether they are one- or two-sided<br>*Only common tests should be described solely by name; describe more complex techniques in the Methods section.*
☒	☐	A description of all covariates tested
☐	☒	A description of any assumptions or corrections, such as tests of normality and adjustment for multiple comparisons
☐	☒	A full description of the statistical parameters including central tendency (e.g. means) or other basic estimates (e.g. regression coefficient) AND variation (e.g. standard deviation) or associated estimates of uncertainty (e.g. confidence intervals)
☐	☒	For null hypothesis testing, the test statistic (e.g. *F*, *t*, *r*) with confidence intervals, effect sizes, degrees of freedom and *P* value noted<br>*Give P values as exact values whenever suitable.*
☒	☐	For Bayesian analysis, information on the choice of priors and Markov chain Monte Carlo settings
☒	☐	For hierarchical and complex designs, identification of the appropriate level for tests and full reporting of outcomes
☒	☐	Estimates of effect sizes (e.g. Cohen's *d*, Pearson's *r*), indicating how they were calculated

Our web collection on statistics for biologists contains articles on many of the points above.

Software and code

Policy information about availability of computer code

Data collection	Phase contrast images were acquired with an EVOS microscope (Advanced microscope group, Bothell, WA). Fluorescent confocal images were acquired with a confocal FV3000 microscope (Olympus LS). Epifluorescent images were acquired with both a Nikon (eclipse E400) and a Keyence microscope (BZ-X810). Nikon acquired images were also processed through the nuance software to generate raw Jpeg images. All images were processed for brightness adjustment and quantification through ImageJ. Single cell RNA sequencing data was obtained using the Chromium 10x platform V2 chemistry kit and the Chromium single cell 3' reagent kit (CG00052 Rev B) to generate cDNA libraries. CDNA libraries were sequenced using the Illumina Hiseq 4000 and FASTQ files were aligned using Cell ranger and mapped against the human reference genome GRCh38. Published sequencing datasets were obtained through UCSC cell browser.
Data analysis	Fluorescent images were analyzed with ImageJ (v 2.0.0). Statistical tests and plots were performed in Prism 8. Single cell RNA sequencing files were processed through both SoptSC pipeline in Matlab and Seurat pipeline (v 4.2) in R studio. Gene enrichment studies (KEGGprofilr v 2.12, ReactomePA v 3.19, topGO v 3.19) were performed in R studio. All codes were obtained from publicly available sources and tutorials.

For manuscripts utilizing custom algorithms or software that are central to the research but not yet described in published literature, software must be made available to editors and reviewers. We strongly encourage code deposition in a community repository (e.g. GitHub). See the Nature Portfolio guidelines for submitting code & software for further information.

Data

Policy information about availability of data

All manuscripts must include a data availability statement. This statement should provide the following information, where applicable:
- Accession codes, unique identifiers, or web links for publicly available datasets
- A description of any restrictions on data availability
- For clinical datasets or third party data, please ensure that the statement adheres to our policy

All single cell RNA sequencing files are uploaded to the NCBI GEO database and are under the GEO accession number GSE296691

Research involving human participants, their data, or biological material

Policy information about studies with human participants or human data. See also policy information about sex, gender (identity/presentation), and sexual orientation and race, ethnicity and racism.

Reporting on sex and gender	Post mortem human choroid plexus tissue was obtained from UCI medical center autopsy service and sectioned by the Experimental tissue resource core facility. 4 samples are male (average age 35.5 pcw) and 3 samples are female (average age 38 pcw with one outlier of 19 years).
Reporting on race, ethnicity, or other socially relevant groupings	Socially relevant groupings were not considered when planning or analyzing these studies.
Population characteristics	Samples had a range post mortem reports: pulm aplasia chorioamnionitis, oligohydramnios, GDM, cyanosis placental abruption pulmonary hemorrhage, likely sepsis/coagulopathy twin A anhydramnios and IUFD, twin B demise at 8w SLE, lupus nephritis, pulmonary fibrosis
Recruitment	No recruitment was done in this study. Samples were obtained from the UCI medical center autopsy service.
Ethics oversight	These studies were all conducted under the approval of the human embryonic stem cell research overview committee (hESCRO) and the Institutional review board protocols approved by UCI.

Note that full information on the approval of the study protocol must also be provided in the manuscript.

Field-specific reporting

Please select the one below that is the best fit for your research. If you are not sure, read the appropriate sections before making your selection.

☒ Life sciences ☐ Behavioural & social sciences ☐ Ecological, evolutionary & environmental sciences

For a reference copy of the document with all sections, see nature.com/documents/nr-reporting-summary-flat.pdf

Life sciences study design

All studies must disclose on these points even when the disclosure is negative.

Sample size	Sample size fore single cell sequencing studies were based on previous publications (PMID: 33932339, PMID: 30923815, PMID: 32527923) where we aimed for ~10,000 cells captured per sample. Number of samples was based on minimum numbers to capture a developmental trajectory. Number of fluorescent images used for quantification at least three images, where each image contained a minimum of ~250 cells) where all cells per image were quantified for a given experiment. No statistics was performed to pre-determine sample size.
Data exclusions	Apart from filtering low quality cells and reads from single cell RNA seq data, no other data was excluded from these studies. Single cell QC metrics were based on previous publications standards (PMID: 34062119, PMID: 33932339, PMID: 30923815, PMID: 32527923).
Replication	Quantification of fluorescent signal in postmortem human tissue used multiple tissue sections across multiple glass slides. When possible, samples from different patients were used. Qualitative assessment of fluorescent signal also involved multiple tissue sections across slides per patient and images were representative across sections. Quantitative studies with derived human CPEC, apart from the TTR ELISA and MTO treatment, were performed with multiple chamber slides in at least three independent CPEC derivations. MTO studies were performed in two independent derivations. TTR ELISA was performed once but involved longitudinal across multiple slides and wells. Movies of cilia motility were performed on cells derived from the same derivation (i.e., from the same initial plating) and are representative of movies imaged from multiple wells and chamber slides. For studies tracking changes across the age of derived CPECs, time points were from the same derivation. Bioinformatic analyses were conducted using multiple redundant approaches.
Randomization	Cell treatment experiments were randomized across the wells of a given slide and across slides. Immunostaining combinations of cell cultures and tissue sections was assigned randomly.
Blinding	All experiments contained a degree of blindness. For image acquisition, channels of interest were kept blind to the user and for quantification, sample information was coded in addition to the user being blind to channels of interest. Users created ROIs using a channel containing a membrane marker and were kept blind to the signal in the remaining channels while the ROIs collected measurements in every channel.

Behavioural & social sciences study design

All studies must disclose on these points even when the disclosure is negative.

Study description	
Research sample	
Sampling strategy	
Data collection	
Timing	
Data exclusions	
Non-participation	
Randomization	

Ecological, evolutionary & environmental sciences study design

All studies must disclose on these points even when the disclosure is negative.

Study description	
Research sample	
Sampling strategy	
Data collection	
Timing and spatial scale	
Data exclusions	
Reproducibility	
Randomization	
Blinding	

Did the study involve field work? ☐ Yes ☐ No

Field work, collection and transport

Field conditions	
Location	
Access & import/export	
Disturbance	

Reporting for specific materials, systems and methods

We require information from authors about some types of materials, experimental systems and methods used in many studies. Here, indicate whether each material, system or method listed is relevant to your study. If you are not sure if a list item applies to your research, read the appropriate section before selecting a response.

Materials & experimental systems

n/a	Involved in the study	
☐	☒	Antibodies
☐	☒	Eukaryotic cell lines
☒	☐	Palaeontology and archaeology
☒	☐	Animals and other organisms
☒	☐	Clinical data
☒	☐	Dual use research of concern
☒	☐	Plants

Methods

n/a	Involved in the study	
☒	☐	ChIP-seq
☒	☐	Flow cytometry
☒	☐	MRI-based neuroimaging

Antibodies

Antibodies used

The following primary antibodies were used: Anti-AE2 (S.C. BIOTECH, SC46710, 1/250, goat), Anti-AQP1 (EMD MILLIPORE, AB2219, 1/1000, rabbit), Anti-ARL13B (PROTEINTECH, 17711-1-AP, 1/1000, rabbit), Anti-ATPB (ABCAM, AB14730, 1/500, mouse), Anti-CLDN1 (INVITROGEN, 717800, 1/500, rabbit), Anti-eea1 (CELL SIGNALING, 2411S, 1/250, rabbit), Anti-Nestin (EMD MILLIPORE, ABD69, 1/500, rabbit), Anti-OTX2 (S.C. BIOTECH, SC133873, 1/100, mouse), Anti-SOX2 (INVITROGEN, PA1-094, 1/300, rabbit), Anti-TTR (ABCAM, AB9015, 1/3000, sheep), Anti-ZO-1 (INVITROGEN, 339100, 1/500, mouse), Anti-FOXN4 (S.C. BIOTECH, sc-377166, 1/500, mouse), Anti-MCIDAS (INVITROGEN, PA5-67092, 1/500, rabbit), Anti-CLDN5 (INVITROGEN, 35-2500, 1/200, mouse), Anti-HTR2C (SINO BIOLOGICAL, 203151-T42, 1/500, rabbit), Anti-ATF5 (S.C. BIOTECH, sc-377168, 1/500, mouse), Anti-SC5D (INVITROGEN, PA5-67092, 1/250, rabbit).
The following secondary antibodies were used: Donkey anti-mouse (Thermo Fisher, A31570, 1/500, 555), Donkey anti-mouse (Thermo Fisher, A10038, 1/500, 680), Donkey anti-mouse (Thermo Fisher, A21202, 1/500, 488), Donkey anti-sheep (Thermo Fisher, A11015, 1/500, 488), Donkey anti-goat (Thermo Fisher, A11055, 1/500, 488), Donkey anti-rabbit (Thermo Fisher, A31572, 1/500, 555), Donkey anti-rabbit (Thermo Fisher, A32754, 1/500, 647)

Validation

All validation was performed by the manufacture and reported on their website, cited studies, or the product summary report sent by the manufacturer.

Eukaryotic cell lines

Policy information about cell lines and Sex and Gender in Research

Cell line source(s)	ESC H1 cell line was obtained from WiCell Research Institute in Madison WI and iPSC ADRC6 cell line was obtained from UCI ADRC iPSC bank. All work with the ESC and iPSC lines were conducted under the approval of the human embryonic stem cell research overview (hESCRO) committees at UCI.
Authentication	Both ESC and iPSC lines underwent immunocytochemistry for pluripotent markers to confirm their pluripotent state.
Mycoplasma contamination	Both ESC and iPSC lines underwent mycoplasma screening using commercially available mycoplasma detection kits routinely every 3 months. All cell lines were negative for mycoplasma.
Commonly misidentified lines (See ICLAC register)	No commonly misidentified lines were used in this study

Palaeontology and Archaeology

Specimen provenance	
Specimen deposition	
Dating methods	

☐ Tick this box to confirm that the raw and calibrated dates are available in the paper or in Supplementary Information.

Ethics oversight	

Note that full information on the approval of the study protocol must also be provided in the manuscript.

Animals and other research organisms

Policy information about studies involving animals; ARRIVE guidelines recommended for reporting animal research, and Sex and Gender in Research

Laboratory animals	
Wild animals	
Reporting on sex	
Field-collected samples	
Ethics oversight	

Note that full information on the approval of the study protocol must also be provided in the manuscript.

Clinical data

Policy information about clinical studies

All manuscripts should comply with the ICMJE guidelines for publication of clinical research and a completed CONSORT checklist must be included with all submissions.

Clinical trial registration	
Study protocol	
Data collection	
Outcomes	

Dual use research of concern

Policy information about dual use research of concern

Hazards

Could the accidental, deliberate or reckless misuse of agents or technologies generated in the work, or the application of information presented in the manuscript, pose a threat to:

No	Yes	
☐	☐	Public health
☐	☐	National security
☐	☐	Crops and/or livestock
☐	☐	Ecosystems
☐	☐	Any other significant area

Experiments of concern

Does the work involve any of these experiments of concern:

No	Yes	
☐	☐	Demonstrate how to render a vaccine ineffective
☐	☐	Confer resistance to therapeutically useful antibiotics or antiviral agents
☐	☐	Enhance the virulence of a pathogen or render a nonpathogen virulent
☐	☐	Increase transmissibility of a pathogen
☐	☐	Alter the host range of a pathogen
☐	☐	Enable evasion of diagnostic/detection modalities
☐	☐	Enable the weaponization of a biological agent or toxin
☐	☐	Any other potentially harmful combination of experiments and agents

Plants

Seed stocks

Novel plant genotypes

Authentication

ChIP-seq

Data deposition

☐ Confirm that both raw and final processed data have been deposited in a public database such as GEO.

☐ Confirm that you have deposited or provided access to graph files (e.g. BED files) for the called peaks.

Data access links
May remain private before publication.

Files in database submission

Genome browser session
(e.g. UCSC)

Methodology

Replicates

Sequencing depth

Antibodies

Peak calling parameters

Data quality

Software

Flow Cytometry

Plots

Confirm that:

☐ The axis labels state the marker and fluorochrome used (e.g. CD4-FITC).

☐ The axis scales are clearly visible. Include numbers along axes only for bottom left plot of group (a 'group' is an analysis of identical markers).

☐ All plots are contour plots with outliers or pseudocolor plots.

☐ A numerical value for number of cells or percentage (with statistics) is provided.

Methodology

Sample preparation	
Instrument	
Software	
Cell population abundance	
Gating strategy	

☐ Tick this box to confirm that a figure exemplifying the gating strategy is provided in the Supplementary Information.

Magnetic resonance imaging

Experimental design

Design type	
Design specifications	
Behavioral performance measures	

Imaging type(s)	
Field strength	
Sequence & imaging parameters	
Area of acquisition	

Diffusion MRI ☐ Used ☐ Not used

Preprocessing

Preprocessing software	
Normalization	
Normalization template	
Noise and artifact removal	
Volume censoring	

Statistical modeling & inference

Model type and settings	
Effect(s) tested	

Specify type of analysis: ☐ Whole brain ☐ ROI-based ☐ Both

Statistic type for inference

(See Eklund et al. 2016)

Correction

Models & analysis

n/a	Involved in the study
☐	☐ Functional and/or effective connectivity
☐	☐ Graph analysis
☐	☐ Multivariate modeling or predictive analysis

Functional and/or effective connectivity

Graph analysis

Multivariate modeling and predictive analysis

This checklist template is licensed under a Creative Commons Attribution 4.0 International License, which permits use, sharing, adaptation, distribution and reproduction in any medium or format, as long as you give appropriate credit to the original author(s) and the source, provide a link to the Creative Commons license, and indicate if changes were made. The images or other third party material in this article are included in the article's Creative Commons license, unless indicated otherwise in a credit line to the material. If material is not included in the article's Creative Commons license and your intended use is not permitted by statutory regulation or exceeds the permitted use, you will need to obtain permission directly from the copyright holder. To view a copy of this license, visit http://creativecommons.org/licenses/by/4.0/
